# Bioaccessibility and bioavailability assessment of microalgae-derived minerals for human nutrition

**DOI:** 10.1016/j.crfs.2026.101302

**Published:** 2026-01-06

**Authors:** Fengzheng Gao, Shilei Chen, Xinyue Zhao, Agon Besimi, Christophe Zeder, Maria J. Barbosa, Ferdinand von Meyenn, Alexander Mathys

**Affiliations:** a Sustainable Food Processing Laboratory, Institute of Food Nutrition and Health, ETH Zurich, Schmelzbergstrasse 9, Zurich, 8092, Switzerland; b Laboratory of Nutrition and Metabolic Epigenetics, Institute of Food, Nutrition and Health, ETH Zurich, Schorenstrasse 16, Schwerzenbach, 8603, Switzerland; c Bioprocess Engineering, AlgaePARC, Wageningen University and Research, P.O. Box 16, Wageningen, 6700 AA, the Netherlands

**Keywords:** Micronutrient deficiency, Iron deficiency, *In vitro* digestion, Caco-2 cell absorption, Sustainable nutritional resources

## Abstract

Microalgae-derived minerals are emerging as sustainable nutrient sources for humans; however, knowledge of their bioaccessibility and bioavailability remains limited. This study systematically evaluated mineral (Fe, Ca, Zn, Mg, Cu, Mn, P, K), carbon (C), and nitrogen (N) content and their bioaccessibility from 9 microalgae samples using standardized INFOGEST 2.0 *in vitro* digestion, combined with a human intestinal epithelial (Caco-2) cell model for iron bioavailability assessment. Total mineral content varied notably (Fe, 72.1–3120.9 mg/kg; Ca, 516.9–24,146.3 mg/kg; Zn, 12.1–282.1 mg/kg; Mg, 379.2–15,245.9 mg/kg). Mineral bioaccessibility exhibited a wide variability; for instance, Fe bioaccessibility ranged from 0.5 % in *Tetraselmis chuii* to 83.4 % in green *Chlorella vulgaris*. The bioaccessibility of Ca (0–82.3 %), Zn (51.9–62.2 %), Cu (7.5–89.8 %), Mg (68.0–92.2 %), Mn (6.75–84.3 %), P (9.7–100 %), and K (91.1–100 %) also showed large interspecies differences, similar for C (12.4–78.1 %) and N (39.5–93.8 %). The iron bioavailability of *Arthrospira platensis* and unlysed *Haematococcus pluvialis* is the highest, comparable to that of FeSO_4_. *Tetraselmis chuii* and *Dunaliella salina* exhibit relatively lower bioavailability; however, their levels remain higher than those found in conventional foods. This study demonstrates the potential of microalgae as an innovative and sustainable food source, offering highly bioaccessible minerals and bioavailable iron for human nutrition. It emphasizes the necessity of evaluating both bioaccessibility and bioavailability to accurately assess the nutritional value of microalgae-derived minerals, thereby informing future dietary applications and nutritional strategies in food science.

## Highlights

• Evaluation of microalgae-derived mineral bioaccessibility and iron bioavailability.
• Biomass mineral content alone does not predict bioavailability.
• Bioaccessibilities of microalgae-derived minerals, C, and N are species dependent.
• Iron bioavailability of *Arthrospira* and *Haematococcus* is comparable to FeSO_4_.

## Graphical abstract

**Figure:**
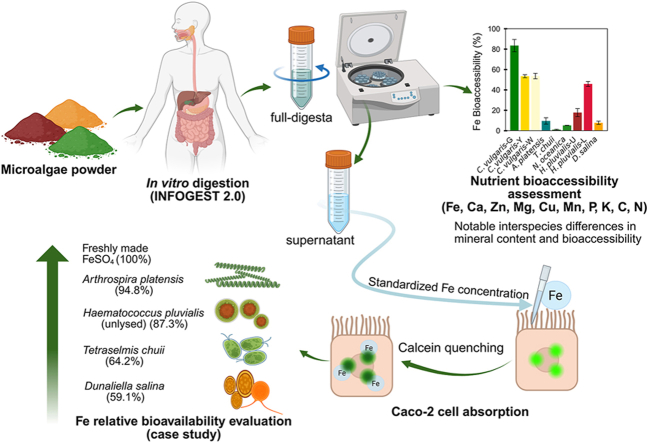

## 1 Introduction

Micronutrient deficiency is a widespread global health problem, primarily driven by inadequate dietary intake. More than 5 billion people are estimated to not consume enough of at least one micronutrient (Passarelli et al., 2024). Among those, mineral deficiencies can be particularly critical, namely, iron, calcium, zinc, and magnesium deficiencies. Iron deficiency is the primary cause of anemia, a condition that affects approximately one in four people globally (Ritchie, 2024). According to the WHO's 2025 Global Anemia estimates, in 2023, 30.7 % of women aged 15–49 suffered from anemia, while in 2019, the prevalence among children aged 6–59 months was 39.8 % (World Health Organization, 2025). Low calcium intake was estimated to be responsible for 3.1 million disability-adjusted life years globally, with older adults being particularly affected due to their increased risk of calcium-related chronic diseases such as colorectal cancer (Ti et al., 2024). Zinc deficiency, affecting around 1.1 billion people, approximately 17.3 % of the world's population, compromises immune function and neurobehavioral development (Kumssa et al., 2015; Wessells and Brown, 2012). Magnesium deficiency is also widespread, with 5–14.5 % of the population affected by subclinical magnesium deficiency, contributing to increased risk of cardiovascular diseases, osteoporosis, and diabetes (DiNicolantonio et al., 2018).

Dietary minerals are typically obtained from a variety of food sources, including animal-based products such as meat, dairy, and seafood, and plant-based foods such as legumes, cereals, nuts, seeds, and leafy vegetables (Solan, 2024). However, reliance on conventional animal-based foods raises concerns related to environmental sustainability, ethical considerations, and dietary preferences (Willett et al., 2019). Although plant-based foods are generally more sustainable, they often have lower minerals content, bioaccessibility, as well as bioavailability due to the presence of absorption inhibitors such as phytates and polyphenols (Rousseau et al., 2020). Given these limitations, the need exists to explore alternative, sustainable, and mineral-dense food sources that can effectively address global mineral deficiencies.

Microalgae are recognized as a rich source of essential minerals, including iron, calcium, zinc, and magnesium, along with proteins, lipids, vitamins, and other bioactive compounds (Koyande et al., 2019; Maroneze et al., 2023). For example, microalgae are a rich source of iron, with reported values of 605 mg/kg dry weight (DW; microalgal DW usually corresponds to about one-third of its fresh weight) in *Nannochloropsis* sp. and 898 mg/kg in *Tetraselmis chuii,* compared to 25.3 mg/kg in beef (fresh weight) (Čmiková et al., 2024; United States Department of Agriculture, 2025). Calcium concentrations in microalgae biomass can range from 1000 mg/kg to 47,300 mg/kg dry weight, with species such as *Tetraselmis chuii* reaching up to 30,000 mg/kg and *Chlorella vulgaris* up to 47,300 mg/kg (Maroneze et al., 2023; Tibbetts et al., 2015; Batista et al., 2013). Magnesium levels in microalgae are similarly high, reported to be between 2720 and 7410 mg/kg, depending on the species (Prates, 2025). Zinc content also varies significantly among microalgal species, with levels ranging from 17 to 232 mg/kg (Maroneze et al., 2023). These findings highlight the dense and diverse mineral profiles of microalgae, positioning them as promising alternatives to address global mineral deficiencies through sustainable food innovations.

Although microalgae are rich in essential minerals and bioactive compounds, a high nutrient content does not necessarily guarantee effective absorption by the human body (Rodrigues et al., 2024). The structural properties of microalgae cells, particularly the composition and rigidity of their cell walls, can limit the release of intracellular nutrients. Many microalgae species, such as *Chlorella* and *Nannochloropsis*, possess rigid cell walls composed of cellulose, hemicellulose, and glycoproteins, which can act as barriers to nutrient absorption (Machado et al., 2022; Scholz et al., 2014). Meanwhile, the thickness and complexity of the cell wall structure vary among species. For instance, compared to *Chlorella*, which has a rigid cell wall composed of cellulose and sporopollenin, *Arthrospira* has a more digestible cell wall composed of peptidoglycan, making nutrients more bioaccessible (Teuling et al., 2017).

To accurately evaluate the digestion potential of nutrients from microalgae, bioaccessibility has emerged as an important indicator (Grundy et al., 2025). In the context of nutritional assessment, bioaccessibility refers to the proportion of nutrients that is released from the food matrix during gastrointestinal digestion and becomes accessible for intestinal absorption (Dima et al., 2020). *In vitro* digestion models, such as the standardized INFOGEST 2.0 protocol, simulate human gastrointestinal digestion to estimate bioaccessibility (Brodkorb et al., 2019). Additionally, *in vitro* cellular models such as the human intestinal epithelial (Caco-2) cell monolayer are widely used to estimate bioavailability, defined as the fraction of a nutrient that is absorbed across the intestinal epithelium and becomes available for physiological functions (Dima et al., 2020; Glahn, 2022).

Despite extensive data on mineral content in microalgae, the comparative mineral bioaccessibility and intestinal absorption potential across different industrialized species remain poorly understood. This gap limits our understanding of whether microalgae can truly serve as nutritionally meaningful mineral sources. Therefore, the purpose of this study is to evaluate and compare the bioaccessibility of essential minerals from 9 microalgae species using the standardized INFOGEST 2.0 protocol, and to investigate the intestinal absorption potential of microalgae-derived iron using polarized human intestinal epithelial (Caco-2) cells (Brodkorb et al., 2019; Glahn, 2022; Wilschefski and Baxter, 2019). This study evaluated 9 different microalgae samples, including *Chlorella vulgaris* (green, yellow, and white), *Arthrospira platensis*, *Tetraselmis chuii*, *Nannochloropsis oceanica*, *Dunaliella salina*, and *Haematococcus pluvialis* (unlysed and lysed). These microalgae were selected because they represent taxonomically diverse microalgae widely used in food and feed applications, and they are all commercially produced under industrial scales (Chen et al., 2024).

The research hypothesis is that microalgae species could show different mineral bioaccessibility and iron bioavailability, potentially influenced by their inherent structural characteristics and cell compositions, which could be highly bioavailable mineral resources compared to conventional foods. The novelty of this work lies in providing the first integrated, species-wide comparison of both mineral bioaccessibility and iron bioavailability across 9 commercially produced microalgae, using a combination of mineral profiling, the standardized INFOGEST 2.0 protocol, and a Caco-2 model. The findings of this study contribute to the knowledge on the nutritional value of microalgae-derived minerals and support applications of microalgae as sustainable, nutritional, and functional food sources.

## 2 Materials and methods

### 2.1 Material

#### 2.1.1 Microalgae samples

The microalgae powder *Chlorella vulgaris* green (*C. vulgaris-*G), *Chlorella vulgaris* yellow (*C. vulgaris*-Y), *Chlorella vulgaris* white (*C. vulgaris*-W), *Arthrospira platensis* (*A. platensis*), *Tetraselmis chuii* (*T. chuii*), and *Nannochloropsis oceanica* (*N. oceanica*) were purchased from Allmicroalgae (Pataias, Portugal). *Haematococcus pluvialis* (unlysed, *H. pluvialis*-U and lysed, *H. pluvialis*-L) were kindly provided by BGG World (Beijing, China). *Dunaliella salina* (*D. salina*) was kindly provided by VIRIDE GmbH (Steinbach, Germany).

#### 2.1.2 Chemicals

The digestive enzymes used in this study were lipase and pepsin from the rabbit gastric extract (RGE, Lipolytech, France), pancreatin (from porcine pancreas, Sigma-Aldrich, USA), and bovine bile (Sigma-Aldrich, USA). RGE and pancreatin were stored at −20 °C, while bile was kept at room temperature. Hydrochloric acid (HCl, 1 M and 6 M) and sodium hydroxide (NaOH, 1 M and 6 M) were prepared in advance and stored at room temperature for pH adjustment during the simulated digestion. The total nitrogen (TN) standard solution (300 mg/L, prepared from NaNO_3_) and the total organic carbon (TOC) standard solution (1000 mg/L, Sigma-Aldrich, Switzerland) were stored at 4 °C. All chemicals and reagents used in this study, together with catalog numbers and manufacturers, are listed in Table S1.

#### 2.1.3 Ash content determination

Ash content was determined using a standard gravimetric method. Approximately 0.3–1.0 g of microalgae powder was weighed into pre-dried ceramic crucibles and incinerated in a muffle furnace at 575 °C for at least 4 h until a constant weight was reached. After cooling in a desiccator, ash percentage was calculated based on the weight difference before and after incineration.

### 2.2 *In vitro* digestion

The *in vitro* digestion process was conducted following the standardized INFOGEST 2.0 protocol (Brodkorb et al., 2019) with modifications to measure the bioaccessibility of nutrients in microalgae samples. The digestion procedure consisted of three sequential phases: oral, gastric, and intestinal digestion, performed under physiological conditions at 37 °C. To ensure the reliability of the results, a blank control (digestive fluids without microalgae) was processed in parallel following the same digestion procedure. This control was used to account for potential background contamination from reagents and digestive enzymes, allowing for appropriate correction during data analysis.

#### 2.2.1 Oral phase

Each microalgae sample (approximately 125 mg, exact weight recorded for each sample) was mixed with 375 μL of Milli-Q water to mimic a swallowable bolus and 400 μL of simulated salivary fluid (SSF), followed by the addition of 3 μL of CaCl_2_ (0.3 M). Milli-Q water was added according to the amount of biomass to achieve a total volume of 1 mL for the bolus. The mixture was vortexed gently to ensure homogeneity and then incubated at 37 °C for 2 min.

#### 2.2.2 Gastric phase

Following oral digestion, 800 μL of simulated gastric fluid (SGF) was added to each sample. The pH was adjusted to 3.0 ± 0.2 using HCl or NaOH. To initiate proteolysis, 100 μL of reconstituted pepsin solution (prepared by dissolving 42.4 mg of pepsin in 900 μL of Milli-Q water) was introduced. Samples were then incubated at 37 °C for 2 h with continuous agitation at 550 rpm using a dry bath (Thermo Scientific, USA). The pH was re-adjusted after 10 and 60 min to maintain stability.

#### 2.2.3 Intestinal phase

For the intestinal phase, 850 μL of simulated intestinal fluid (SIF) was added to each sample. 4 μL of CaCl_2_ (0.3 M) was supplemented, and the pH was adjusted to 7.0 ± 0.2. Bile extract (250 μL, 90.5 mg dissolved in 2.25 mL SIF) and pancreatin solution (500 μL, 425.4 mg dissolved in 4.5 mL SIF) were added sequentially. The mixture was incubated at 37 °C for 2 h, with pH adjustments performed at 10 and 60 min.

#### 2.2.4 Post-digestion processing

After the digestion was completed, 1.5 mL of the total digest was collected in acid-washed tubes for subsequent analysis. The remaining digesta was centrifuged at 10,000 *g*, 4 °C for 30 min to separate the bioaccessible fraction (supernatant) from the undigested residues. The supernatant was transferred to pre-labeled 15 mL conical tubes and stored at −20 °C for further analysis.

### 2.3 Content and bioaccessibility assessment

#### 2.3.1 Mineral analysis

The mineral content of the microalgae samples was quantified using quadrupole inductively coupled plasma mass spectrometry (ICP-MS, iCap RQ, Thermo Scientific, USA). All glassware and sample containers were acid-washed before use, and all sample preparation steps were conducted in a clean room to avoid contamination. Ultrapure water specifically purified for trace element analysis (18.2 MΩ cm, ICP-grade) was used for all sample dilutions and reagent preparation.

For each sample, a 200 μL aliquot was taken from either the full digesta (collected directly after *in vitro* digestion) or the supernatant (obtained after centrifugation of the remaining digesta), both derived initially from 125 mg of microalgae powder as described in Section 2.2. The aliquots were transferred into Teflon digestion tubes, and 4 mL of 65 % (v/v) nitric acid (HNO_3_) was added to each tube. The vessels were sealed and subjected to microwave-assisted digestion using a TurboWave system (MLS, Germany), following a controlled temperature and pressure program. After digestion, the samples were cooled down to room temperature before being transferred to pre-weighed tubes. Each digested solution (initial volume 4.2 mL) was diluted with ultrapure water to approximately 50 mL, and the final weight after dilution was recorded. The net weight of the diluted solution was calculated by subtracting the weight of the empty tube. This value was used as an estimate of the sample volume for subsequent data analysis.

The diluted samples were introduced into the ICP-MS system via an autosampler, and elemental concentrations were determined under optimized conditions. A multi-element calibration curve was prepared using certified ICP-MS standards (Inorganic Ventures, USA) at various concentrations. The analysis was performed in kinetic energy discrimination mode with helium as collision gas to minimize polyatomic interferences. Internal standards (e.g., scandium, rhodium, indium) were used to correct for instrumental drift and matrix effects.

All analytes (Mg, P, K, Ca, Mn, Fe, Cu, Zn) were quantified using a five-point multi-element calibration standard (Inorganic Ventures, USA), with concentration ranges of 20–300 ppb (Mg, Zn), 1–15 ppm (P), 200–3000 ppb (K, Ca), 12.5–200 ppb (Mn), 100–1500 ppb (Fe), 3.125–50 ppb (Cu). Calibration curves were generated by least-squares linear regression, and all elements showed excellent linearity (R^2^ ≥ 0.99). Internal standards (Sc, Rh, In) corrected for drift and matrix effects, and quantification was performed using Qtegra software (Thermo Scientific, USA).

#### 2.3.2 Total organic carbon and total nitrogen analysis

Supernatant and full digesta (300 μL) were transferred into 20 mL pre-cleaned total organic carbon (TOC) borosilicate glass vials. Each sample was then diluted 50-fold with Milli-Q water to reach a final volume of 15 mL. The final TOC and total nitrogen (TN) concentrations were calculated by applying the 50 × dilution factor to reflect the original sample concentrations in the full digesta and supernatant.

Calibration curves for C (50–600 mg/L) and N (10–100 mg/L) were prepared using TOC standard and sodium nitrate (NaNO_3_) as standard solutions. Each standard was also diluted with Milli-Q water to match the final sample matrix.

The TOC and TN were analyzed using a TOC/TN analyzer (TOC/TN, Shimadzu, Japan). The instrument operates based on catalytic combustion (680 °C), followed by Non-Dispersive Infrared (NDIR) detection for TOC and chemiluminescence detection for TN. For TOC, the NPOC (Non-Purgeable Organic Carbon) method was used, where samples were acidified to remove inorganic carbon before measurement (Shimadzu a). For TN, the total nitrogen bound in the sample was converted to nitrogen oxides and detected by a chemiluminescence detector (Shimadzu b). The resulting data were used to quantify the total organic carbon and nitrogen content in the digested samples, following correction for dilution. All major equipment used throughout the analytical procedures is summarized in Table S2.

### 2.4 Iron bioavailability determination

#### 2.4.1 Solutions in bioavailability measurement

Complete and low-iron MEM (Minimal Essential Medium) media were used for cell culture and iron absorption experiments. The complete MEM medium contained 20 % FBS (Fetal Bovine Serum), 1 % MEM Non-Essential Amino Acids (NEAA, 100 × , Gibco), and 1 % Pen-Strep (100 U/mL penicillin and 100 μg/mL streptomycin, Gibco). The low-iron medium was prepared by reducing the FBS concentration from 20 % to 2 %. A 1 mM Calcein-AM solution was prepared in 1 × HBSS (Hanks’ Balanced Salt Solution) and stored at −20 °C for use in the absorption assay. The FeSO_4_ standard solution was prepared freshly before each measurement at a final concentration of 3 ppm (53.6 μM). All other required solutions, including PBS (pH 7.4), Trypsin-EDTA (0.25 %), Tyrode solutions (pH 5.5 and 7.0), collagen (1:500), and HBSS, were either commercially available or prepared according to standard protocols.

#### 2.4.2 Caco-2 cell culture

Caco-2 cells (passage 5) were thawed and cultured at 37 °C in a humidified incubator with 5 % CO_2_. Cells were seeded in 24-well plates pre-coated with 1:500 collagen at a density of 50,000 cells/cm^2^. The culture medium was refreshed every two days. After 12 days of differentiation, the cell monolayers were used for the iron absorption assay (Glahn, 2022).

#### 2.4.3 Kinetic iron absorption

The medium was replaced with low-iron MEM 24 h before measurement to induce iron deficiency. On day 13, the cells were washed with 1 × HBSS and incubated with 300 μL of 1 mM Calcein-AM solution at 37 °C for 30 min. Excess extracellular dye was removed by washing the cells three times with 0.5 mL HBSS.

Supernatants of digested microalgae (obtained from *in vitro* digestion) were thawed and applied immediately. The FeSO_4_ reference and microalgae samples were diluted to a normalized iron concentration of 2.68 μM, based on ICP-MS results. Each sample was added to the wells immediately before measurement. Fluorescence (excitation at 485 nm, emission at 530 nm) was monitored every 2 min for 90 min at 37 °C using a plate reader (SynergyMx, BioTek Instruments GmbH, Switzerland). The fluorescence decline over time was calculated to estimate cellular iron uptake.

### 2.5 Data analysis

#### 2.5.1 Bioaccessibility determination

Nutrient bioaccessibility was defined as the proportion of nutrients released into the supernatant (bioaccessible fraction) relative to the total nutrient content in the full digesta, and the results are expressed as a percentage (%) according to Equation (1).

(1) $$Bioaccessibility\left(\%\right)=\left({Con.}_{\text{Supernatant}}/{Con.}_{\text{Full}\;\text{Digesta}}\right)\times 100\%$$

Where ${Con.}_{\text{Supernatant}}$ denotes nutrient concentration in the sample supernatant (mg/L, measured by ICP-MS or TOC/TN), ${Con.}_{\text{Full}\;\text{Digesta}}$ denotes nutrient concentration in the full digesta (mg/L).

#### 2.5.2 Biomass nutrient content

The total nutrient content of microalgae (biomass) was determined from the full digesta concentration using Equation (2), representing the complete nutrient content in the sample before digestion.

(2) $$Biomass\;nutrient\;content\;\left(mg/kg\right)=\frac{\left({Con.}_{\text{Full}\;\text{Digesta}}\times V\right)}{M}$$

Where V denotes the total volume of the digestion fluid (4 mL, i.e., 0.004 L), M denotes the mass of microalgae sample used for digestion (kg, individually recorded; g was used for C and N calculation with unit of mg/g in the content).

#### 2.5.3 Bioaccessible nutrient content

The absolute amount of bioaccessible nutrients was determined by multiplying the bioaccessibility (%) by the biomass nutrient content, as shown in Equation (3).

(3) Bioaccessiblecontent(mg/kg)=bioaccessibility×biomassnutrientcontent

All nutrient analyses were conducted according to Section 2.3. For minerals, the biomass nutrient content was calculated in mg/kg, while mg/g for C and N, due to the large values.

#### 2.5.4 Calculation of iron bioavailability

Iron uptake was quantified using Calcein-AM, a fluorescent probe whose signal is quenched when iron is absorbed into Caco-2 cells (Ma et al., 2015). In this study, the fluorescence signal stabilized at 30 min for FeSO_4_ and most microalgae samples; therefore, the 30-min time point was used to calculate iron bioavailability.

The percentage decline in fluorescence at 30 min was calculated relative to the baseline fluorescence at time 0 ($F_{0}$), as shown in Equation (4):

(4) $$Percentage\;decline=\left[\left(F-F_{0}\right)/F_{0}\right]\times 100\%$$

Where, $F_{0}$ denotes the fluorescence intensity at time 0, and F is the fluorescence at the real time. All measurements were performed with the same autogain setting (119).

The relative bioavailability of iron in the sample compared to the reference FeSO_4_ was calculated as shown in Equation (5):

(5) $$Relative\;bioavailability=\left({Sample}_{percentage\;decline}/{FeSO_{4}}_{percentage\;decline}\right)\times 100\%$$

#### 2.5.5 Statistical analysis

For mineral bioaccessibility, bioaccessible content, and C/N measurements, three independent *in vitro* digestions were performed (*n* = 3). For iron bioavailability, each digest batch was analyzed with 3–5 experimental replicates in triplicate wells, resulting in 9–15 measurements per species. Specifically, the number of measurements was as follows: *C. vulgaris*-G (*n* = 12), *C. vulgaris*-Y (*n* = 15), *C. vulgaris*-W (*n* = 12), *A. platensis* (*n* = 15), *T. chuii* (*n* = 9), *N. oceanica* (*n* = 15), *H. pluvialis*-U (*n* = 9), *H. pluvialis*-L (*n* = 15), and *D. salina* (*n* = 9).

Fluorescence kinetic data from the SynergyMx plate reader were processed using Gen5™ Data Analysis Software (BioTek Instruments, USA). All statistical analyses were performed using Microsoft Excel (version 2024, Microsoft Corp., USA). Comparisons between each microalgal sample and the FeSO_4_ reference were made using Welch's *t*-test. A significance level of *p* < 0.05 was applied for all analyses. All figures were generated using Python (version 3.12, Python Software Foundation, USA) via custom scripts for data visualization.

## 3 Results and discussion

### 3.1 Biomass composition of minerals

**Fig. 1:**
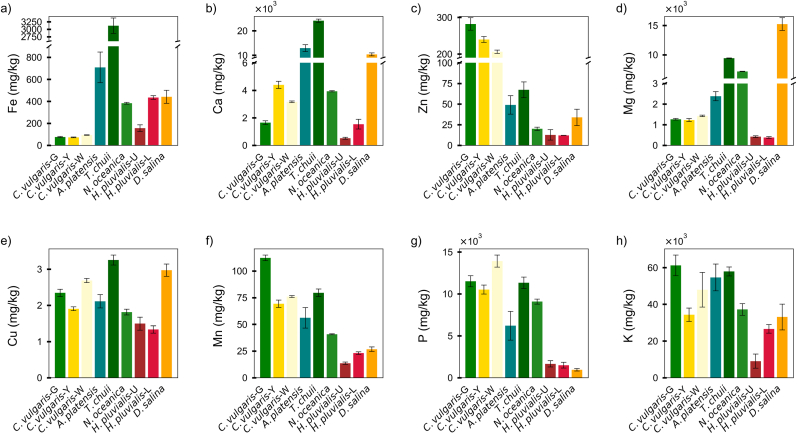
Mineral content (mg/kg) in different microalgae species. (a) Fe, (b) Ca, (c) Zn, (d) Mg, (e) Cu, (f) Mn, (g) P, (h) K. ***Note****: From left to right, the abbreviations represent the following species: Chlorella vulgaris green (C. vulgaris-G); Chlorella vulgaris yellow (C. vulgaris-Y); Chlorella vulgaris white (C. vulgaris-W); Arthrospira platensis (A. platensis); Tetraselmis chuii (T. chuii); Nannochloropsis oceanica (N. oceanica); Haematococcus pluvialis (unlysed, H. pluvialis-U); Haematococcus pluvialis (lysed, H. pluvialis-L); and Dunaliella salina (D. salina). Data are presented as mean ± SD (n = 3).*

To assess the nutritional potential of various microalgal strains, the content of 8 essential minerals, namely Fe, Ca, Zn, Mg, Cu, Mn, P, and K, was measured (Fig. 1 and Table S3). To better understand the biomass composition profile, the ash content of each species was determined to provide an overview of their total inorganic fraction. Ash contents ranged from 2.1 % to 52.7 % depending on the species, and the full dataset is presented in Table S4.

Fe content varied widely among the 9 microalgal samples, ranging from 72.1 ± 3.2 mg/kg in *C. vulgaris*-Y to 3120.9 ± 255.6 mg/kg in *T. chuii* (Fig. 1a). Relatively lower Fe concentrations were observed in *C. vulgaris*-Y (72.1 ± 3.2 mg/kg), *C. vulgaris*-G (75.9 ± 5.2 mg/kg), and *C. vulgaris*-W (93.5 ± 2.2 mg/kg), all below 100 mg/kg. Despite being at the lower end of the tested species, these values still exceeded those found in conventional foods such beef (25.6 mg/kg), chicken (4.9 mg/kg), or tuna fish (9.4 mg/kg) (Iaquinta et al., 2023). Higher levels were detected in *H. pluvialis*-U (156.0 ± 30.2 mg/kg) and *H. pluvialis*-L (434.5 ± 16.8 mg/kg), followed by *D. salina* (441.8 ± 59.2 mg/kg), *N. oceanica* (382.8 ± 8.3 mg/kg), and *A. platensis* (709.2 ± 138.9 mg/kg). The highest Fe content was found in *T. chuii* (3120.9 ± 255.6 mg/kg), almost 22times higher than the Fe content of soybean seed (143.0 mg/kg) (Zewudie and Gemede, 2024).

Among the microalgae tested, *T. chuii* exhibited the highest Ca content (24,146.3 ± 664.4 mg/kg), followed by *A. platensis* (12,920.8 ± 1416.6 mg/kg) and *D. salina* (10,359.6 ± 587.6 mg/kg) (Fig. 1b). *C. vulgaris*-G (1651.7 ± 133.6 mg/kg), *H. pluvialis*-L (1537.3 ± 353.8 mg/kg), and *H. pluvialis*-U (516.9 ± 69.1 mg/kg) presented relatively lower contents. Notably, even the lowest Ca content in *H. pluvialis*-U (516.9 mg/kg) was close to that of the whole egg (Cortés-Herrera et al., 2023), and nearly double that of plain tofu (275 mg/kg) (Paz et al., 2021). Moreover, most samples exceeded the Ca content reported for soybean seeds (1742 - 2688 mg/kg) (Zewudie and Gemede, 2024). These results reinforce the rich mineral profile of microalgae and highlight their promise as alternative calcium sources.

Zn content showed substantial interspecies variation among the 9 microalgae (Fig. 1c). The highest Zn concentration was observed in *C. vulgaris*-G (282.1 ± 16.9 mg/kg), suggesting a genus-specific capacity for Zn accumulation. In contrast, markedly lower contents were observed in *N. oceanica* (20.0 ± 2.0 mg/kg), *H. pluvialis*-U (12.7 ± 6.3 mg/kg), and *H. pluvialis*-L (12.1 ± 0.01 mg/kg). Even the lowest Zn value among the microalgae (*H. pluvialis*-L) exceeded that of many commonly consumed foods such as tuna fish (10.4 mg/kg), soybean (10.6 mg/kg), and quinoa (4.8 mg/kg) (Iaquinta et al., 2023). Furthermore, *C. vulgaris*-G exhibited a Zn content over three times that of milk powder (86.2 mg/kg), highlighting its potential as a rich dietary Zn source (Cortés-Herrera et al., 2023).

*H. pluvialis*-U (423.0 ± 41.8 mg/kg) and *H. pluvialis*-L (379.2 ± 43.9 mg/kg) exhibited relatively lower Mg content (Fig. 1d). Three *C. vulgaris* variants presented intermediate values, with *C. vulgaris*-W at 1427.8 ± 36.5 mg/kg, *C. vulgaris*-G at 1264.5 ± 37.9 mg/kg, and *C. vulgaris*-Y at 1228.2 ± 72.5 mg/kg. The Mg content in *C. vulgaris* was comparable to or slightly higher than commonly consumed foods such as beef (726 mg/kg), milk powder (1089.7 mg/kg), and tomato (1363 mg/kg) (Cortés-Herrera et al., 2023; Menezes et al., 2018). Higher Mg content was observed in *N. oceanica* (7198.1 ± 32.9 mg/kg), followed by *T. chuii* (9436.7 ± 47.3 mg/kg), while *D. salina* displayed the highest Mg content (15,245.9 ± 1073.0 mg/kg). These results indicate a strong species dependence in Mg accumulation capacity among microalgae.

In addition to these minerals associated with micronutrient deficiencies, Cu, Mn, P, and K were also measured to provide a more comprehensive mineral profile of the microalgae. These elements are essential cofactors in various metabolic and enzymatic processes, and their quantification allows for a better understanding of the overall nutritional potential of each species (Jomova et al., 2022). Cu levels were low in all species, with *T. chuii* having the highest concentration at 3.3 ± 0.1 mg/kg, while *H. pluvialis*-L contained the lowest at 1.3 ± 0.1 mg/kg (Fig. 1e). Mn concentrations varied more significantly, with *C. vulgaris*-G exhibiting the highest value (112.3 ± 2.7 mg/kg) and *H. pluvialis*-U the lowest (13.6 ± 1.1 mg/kg) (Fig. 1f). P was found in the highest amounts in *C. vulgaris*-W (13,917.9 ± 720.7 mg/kg) (Fig. 1g), whereas *D. salina* had the lowest concentration of 942.6 ± 147.2 mg/kg. K was abundant in all species, with *C. vulgaris*-G reaching the highest level (61,235.9 ± 5627.9 mg/kg) and *H. pluvialis*-U showing the lowest at 9042.1 ± 3869.1 mg/kg (Fig. 1h). While these minerals are less commonly associated with deficiencies compared to Fe, Ca, Zn, and Mg, their presence in microalgae still contributes to overall nutritional value.

### 3.2 Bioaccessibility of minerals

**Fig. 2:**
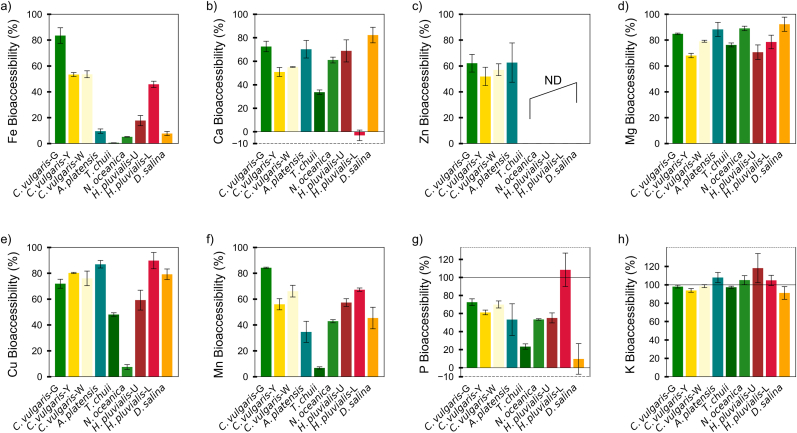
Bioaccessibility (%) of minerals in different microalgae species. (a) Fe, (b) Ca, (c) Zn, (d) Mg, (e) Cu, (f) Mn, (g) P, (h) K. ***Note****: From left to right, the abbreviations represent the following species: Chlorella vulgaris green (C. vulgaris*-G*); Chlorella vulgaris yellow (C. vulgaris-Y); Chlorella vulgaris white (C. vulgaris-W); Arthrospira platensis (A. platensis); Tetraselmis chuii (T. chuii); Nannochloropsis oceanica (N. oceanica); Haematococcus pluvialis (unlysed, H. pluvialis-U); Haematococcus pluvialis (lysed, H. pluvialis-L); and Dunaliella salina (D. salina). ND: Not detected. Data are presented as mean ± SD (n = 3).*

Fe bioaccessibility varied widely among the tested species (Fig. 2a and Table S5), and this variation did not correspond to the total Fe content. For example, *T. chuii* showed an extremely low Fe bioaccessibility (0.51 ± 0.03 %), despite its highest Fe content (3120.9 ± 255.6 mg/kg, Fig. 1a). In contrast, *C. vulgaris*-G, which had one of the lowest Fe contents (75.9 ± 5.2 mg/kg), showed the highest bioaccessibility (83.4 ± 6.0 %). The apparent disconnect suggests that high Fe content in the biomass does not necessarily translate into high bioaccessibility. The low bioaccessibility in *T. chuii* may be due to Fe binding with other compounds, such as polyphenols, dietary fibers, or phytic acid, which could hinder its solubility (Rousseau et al., 2020). Similarly, *N. oceanica* (5.1 ± 0.2 %) and *D. salina* (7.7 ± 1.6 %) also exhibited low Fe bioaccessibility despite moderate total Fe contents. In addition, commercial microalgae biomass is usually not washed during harvest, which may have led to overestimation of non-bioaccessible surface-bound minerals due to biosorption (Spain et al., 2021).

When compared with conventional iron sources, thermally treated lamb meat exhibited Fe bioaccessibility values between 4 and 19 %, depending on the cooking methods (de Higuera et al., 2021a), and beef Fe bioaccessibility was around 29 % (Iaquinta et al., 2023). Plant-based foods such as soybean and quinoa showed Fe bioaccessibility of approximately 41 % and 39 %, respectively (Iaquinta et al., 2023). In this context, the exceptionally high Fe bioaccessibility observed in *C. vulgaris*-G (83 %) is higher than both animal-derived and plant-derived sources, highlighting its potential as a novel iron source.

Ca bioaccessibility displayed a broad range across the tested microalgae species (Fig. 2b and Table S5). The highest Ca bioaccessibility was observed in *D. salina* (82.3 ± 6.6 %), followed by *C. vulgaris-*G (72.5 ± 4.4 %) and *A. platensis* (70.2 ± 7.5 %). In contrast, *T. chuii* (33.7 ± 1.9 %) and *C. vulgaris*-Y (50.9 ± 3.8 %) exhibited relatively lower Ca bioaccessibility. Of note, the negative bioaccessibility meassuered for *H. pluvialis*-L (−3.2 ± 4.5 %), can likely be attributed to the formation of calcium-phosphate precipitates in the digestive environment. According to previous studies on simulated intestinal fluid (SIF) and gastric fluid (SGF), Ca^2+^ ions bind with phosphate (HPO_4_^2−^) to form calcium phosphate (Ca_3_(PO_4_)_2_) precipitates, reducing the measurable soluble Ca fraction during digestion (Wang et al., 2023; Perego et al., 2015). The negative measured value suggests that in *H. pluvialis*-L, a large fraction of Ca was bound in insoluble complexes rather than being in a bioaccessible form.

In comparison, Ca bioaccessibility in beef, chicken, and pork has been reported to range from 8 % to 30 % depending on cooking method (Menezes et al., 2018), while dairy-based fortified matrices reached only 20 %–36 % using the INFOGEST model (Lorieau et al., 2018). These findings highlight the potential of specific microalgae species, such as *D. salina*, *C. vulgaris-G*, and *A. platensis*, to serve as highly efficient, non-dairy calcium sources for populations at risk of Ca deficiency, including individuals following plant-based diets.

Zn bioaccessibility exhibited large differences between species (Fig. 2c and Table S5). Among the samples with detectable Zn, *A. platensis* had the highest Zn bioaccessibility (62.2 ± 4.5 %), followed by *C. vulgaris*-G (62.1 ± 6.8 %), *C. vulgaris*-W (57.1 ± 4.6 %), and *C. vulgaris*-Y (51.9 ± 7.1 %). Compared to conventional foods, Zn bioaccessibility in *A. platensis* and three *C. vulgaris* strains (51.9 %–62.2 %) was lower than that of quinoa (92 %), soybean (87 %), and chicken (69 %), yet still falls within the range of lentils (67 %) and tuna (66 %) (Iaquinta et al., 2023). In contrast, Zn bioaccessibility was below the detection limit in *T. chuii*, *N. oceanica*, *H. pluvialis*-U, *H. pluvialis*-L, and *D. salina*. As shown in Section 3.1, these species already exhibited low Zn concentrations in their biomass. As a result, even if part of the Zn was released during digestion, the absolute amount in the digested fluid might still fall below the detection limit, leading to an apparent lack of bioaccessibility.

Cu, Mg, and K generally showed high bioaccessibility across most species (Fig. 2e–d, h). Cu bioaccessibility remained relatively high in most species, such as *C. vulgaris*-G, *C. vulgaris*-W, *A. platensis*, and *H. pluvialis*-L, exhibiting levels exceeding 48 %, except for *N. oceanica* (7.5 ± 1.9 %). Conventional foods such as soybeans and beef demonstrated Cu bioaccessibility levels around 87 % and 43 %, respectively (Iaquinta et al., 2023). Mg bioaccessibility ranged from 68.0 ± 1.7 % in *C. vulgaris*-Y to 92.2 ± 5.5 % in *D. salina*, with most species exceeding 75 %, suggesting that Mg remains highly bioaccessible in microalgae. In contrast, Mg bioaccessibility in cooked lamb meat was reported between 41 % and 54 %, which remains lower than the values observed in most microalgae species (de Higuera et al., 2021a). K bioaccessibility remained consistently high, exceeding 91 % in nearly all species. This is particularly noteworthy given that lamb meat only exhibited K bioaccessibility in the range of 64–76 % (de Higuera et al., 2021a). The exceptionally high release efficiency in microalgae highlights their potential as K-rich dietary components.

Mn and P exhibited differences in bioaccessibility among microalgae species (Fig. 2f and g). Mn bioaccessibility ranged from 6.8 ± 0.9 % (*T. chuii*) to 84.3 ± 0.5 % (*C. vulgaris*-G), indicating substantial variation in Mn release. Notably, several conventional food sources, including beef and plant-based burgers, exhibited considerably lower Mn bioaccessibility. For example, Mn release from commercial vegetable burgers ranged from 2-29 %, depending on the formulation and thermal processing (de Higuera et al., 2021b). In this context, certain microalgae species demonstrate a clear advantage in delivering bioaccessible Mn. P bioaccessibility was highest in *H. pluvialis*-L (108.4 ± 18.6 %) but significantly lower in *D. salina* (9.6 ± 17.0 %), showing a broad range among species (Fig. 2h). These results compare favorably with cooked lamb meat, which showed P bioaccessibility between 48 and 59 % across cooking methods, further demonstrating the nutritional relevance of select microalgal species (de Higuera et al., 2021a).

In this study, substantial inter-species differences in mineral bioaccessibility were observed. While the exact mechanisms underlying these differences remain to be fully elucidated, several plausible explanations can be proposed based on the known structural and biochemical characteristics of microalgae. First, variations in cell-wall architecture (including thickness, rigidity, and biochemical composition) may influence the extent to which intracellular minerals are released during digestion (González-Hourcade et al., 2023). Species with thick, multi-layered, and polymer-rich cell walls, such as *C. vulgaris* and *N. oceanica*, may exhibit reduced mineral bioaccessibility (Weber et al., 2022; Khamila et al., 2025), because these structures hinder enzymatic and physicochemical disruption during digestion. Second, the presence of mineral-binding compounds such as polyphenols, or phytic acid could limit mineral solubilization by forming insoluble complexes (Kumar et al., 2010; Martínez-Ruiz et al., 2025). Phytic acid acts as a highly negatively charged chelator that forms stable, poorly soluble mineral-phytate complexes across gastrointestinal pH ranges, while polyphenols may additionally bind minerals or promote co-precipitation, collectively reducing mineral bioaccessibility (Kumar et al., 2010). Third, minerals can occur in different intracellular chemical forms (speciation) and subcellular storage pools in microalgae (e.g., polyphosphate-/phosphate-rich granules or different redox states), which may strongly influence their dissolution under gastrointestinal conditions and thus the measurable soluble (bioaccessible) fraction (Sanz-Luque et al., 2020; Gao et al., 2021). These potential factors together offer a reasonable explanation for the species-specific differences observed in this work.

Previous work has also shown that nutrient bioaccessibility in microalgae can be enhanced through cell-disruption techniques such as high-pressure homogenization (HPH) or pulsed electric field (PEF), which increase cell permeability and promote nutrient release (Canelli et al., 2022). Optimizing cultivation conditions (e.g., heterotrophic growth, modulation of light or nutrient stress) and reducing surface-bound minerals via appropriate washing procedures have also been proposed as universal strategies to increase mineral bioaccessibility across diverse microalgal species (Gao et al., 2026).

### 3.3 Bioaccessible content of minerals

**Fig. 3:**
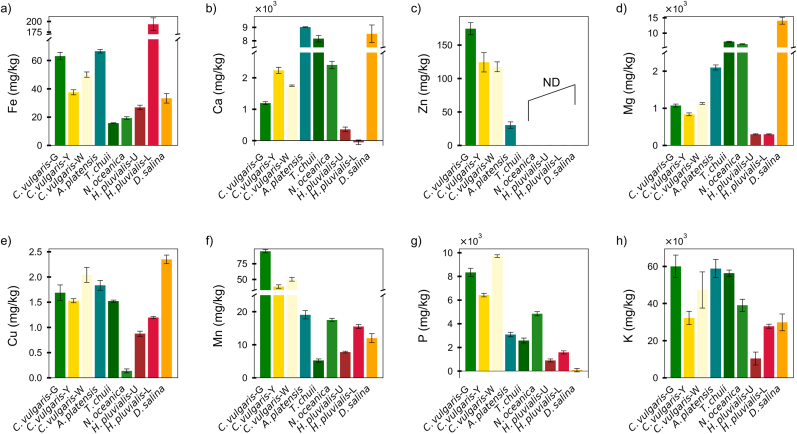
Bioaccessible mineral content (mg/kg) in different microalgae species. (a) Fe, (b) Ca, (c) Zn, (d) Mg, (e) Cu, (f) Mn, (g) P, (h) K. ***Note****: From left to right, the abbreviations represent the following species: Chlorella vulgaris green (C. vulgaris-G); Chlorella vulgaris yellow (C. vulgaris-Y); Chlorella vulgaris white (C. vulgaris-W); Arthrospira platensis (A. platensis); Tetraselmis chuii (T. chuii); Nannochloropsis oceanica (N. oceanica); Haematococcus pluvialis (unlysed, H. pluvialis-U); Haematococcus pluvialis (lysed, H. pluvialis-L); and Dunaliella salina (D. salina). ND: Not detected. Data are presented as mean ± SD (n = 3).*

The bioaccessible mineral contents varied markedly among the 9 microalgae biomass, reflecting a combination of biomass mineral concentration and species-specific bioaccessibility (Fig. 3 and Table S6). Despite moderate or even low total Fe content, some microalgae exhibited notably high bioaccessible iron concentrations. For example, *H. pluvialis*-L exhibited the highest bioaccessible Fe concentration (199.0 ± 10.7 mg/kg), due to its relatively high bioaccessibility (45.8 %). Similarly, *C. vulgaris*-G had a low total Fe content (75.9 mg/kg), yet achieved a high bioaccessible Fe value (63.3 ± 4.6 mg/kg) owing to its exceptional bioaccessibility (83.4 %). In contrast, *T. chuii* exhibited the highest total Fe level (3120.9 mg/kg), but the lowest bioaccessible fraction (16.1 ± 1.0 mg/kg), because of extremely limited Fe bioaccessibility (0.5 %). These observations further support that high total mineral content alone does not necessarily guarantee high nutritional value. Fe bioaccessibility was found to decrease with increased iron in the biomass and could be manipulated by different production modes (Gao et al., 2026). Compared to conventional iron sources such as tofu (4.4 mg/kg), soybean (13.4 mg/kg), smoked salmon (2.3 mg/kg) and tuna (7.2 mg/kg) (Iaquinta et al., 2023; Gao et al., 2026), the bioaccessible Fe content of several microalgae species remains substantially higher, highlighting their potential as effective and sustainable dietary iron sources.

In terms of Ca, the highest bioaccessible contents were detected in *A. platensis* (9069.1 ± 969.1 mg/kg), *D. salina* (8521.8 ± 684.8 mg/kg), and *T. chuii* (8130.1 ± 449.1 mg/kg), attributed to their substantial biomass Ca concentrations coupled with favorable bioaccessibility (>70 %). Conversely, *H. pluvialis*-L exhibited negative bioaccessible Ca, likely due to precipitation reactions forming insoluble calcium-phosphate complexes during digestion. Bioaccessible Zn content was detected exclusively in *A. platensis* (30.5 ± 2.2) and three *C. vulgaris* (117.7–175.1 mg/kg). The bioaccessible Zn content of commonly consumed animal and plant-based foods, such as beef (36.1 mg/kg), soybean (9.2 mg/kg), and quinoa (4.4 mg/kg), has been reported to be substantially lower than that of the three *C. vulgaris* (Iaquinta et al., 2023). For the other species, including *T. chuii*, *D. salina*, *N. oceanica*, and both *H. pluvialis* samples, Zn was below the detection limit after digestion.

Mg exhibited generally high bioaccessible content across different microalgae, particularly in *D. salina* (14,059.8 ± 838.5 mg/kg), *T. chuii* (7191.9 ± 147.2 mg/kg), and *N. oceanica* (6408.5 ± 123.8 mg/kg), due to high bioaccessibility (>76 %). *H. pluvialis*-U and *H. pluvialis*-L displayed the lower values, with bioaccessible Mg contents of 298.5 ± 23.9 mg/kg and 297.6 ± 20.0 mg/kg, respectively. Animal-derived foods such as lamb meat typically provide 220–360 mg/kg of bioaccessible Mg, depending on cooking methods, which is considerably lower than the levels observed in most microalgae (de Higuera et al., 2021a). Among the other measured minerals, *C. vulgaris*-G consistently exhibited the highest bioaccessible content for Mn (94.6 ± 0.6 mg/kg), P (8343.7 ± 428.2 mg/kg), and K (59992.9 ± 943.0 mg/kg). Except for *N. oceanica* (0.14 ± 0.03 mg/kg), the remaining 8 microalgae species exhibited bioaccessible Cu contents ranging from 0.88 ± 0.11 mg/kg in *H. pluvialis*-U to 2.35 ± 0.12 mg/kg in *D. salina*, showing an advantage over beef (0.47 mg/kg) and chicken (0.20 mg/kg) (Iaquinta et al., 2023).

Collectively, these results emphasize the critical role of bioaccessibility in determining the nutritional relevance of microalgal biomass. Although *A. platensis* emerged as a relatively balanced source of multiple minerals, targeted nutritional applications for populations at risk of specific mineral deficiencies (e.g., iron deficiency, zinc deficiency, or individuals with lactose intolerance requiring non-dairy Ca sources) might benefit more specifically from the high Fe content of *H. pluvialis*-L, the Mg-rich biomass of *D. salina* and *T. chuii*, or the superior Zn levels provided by *C. vulgaris*-G. Such distinctions highlight the necessity for species-specific selection based on intended nutritional targets.

### 3.4 Carbon and nitrogen in microalgae

**Fig. 4:**
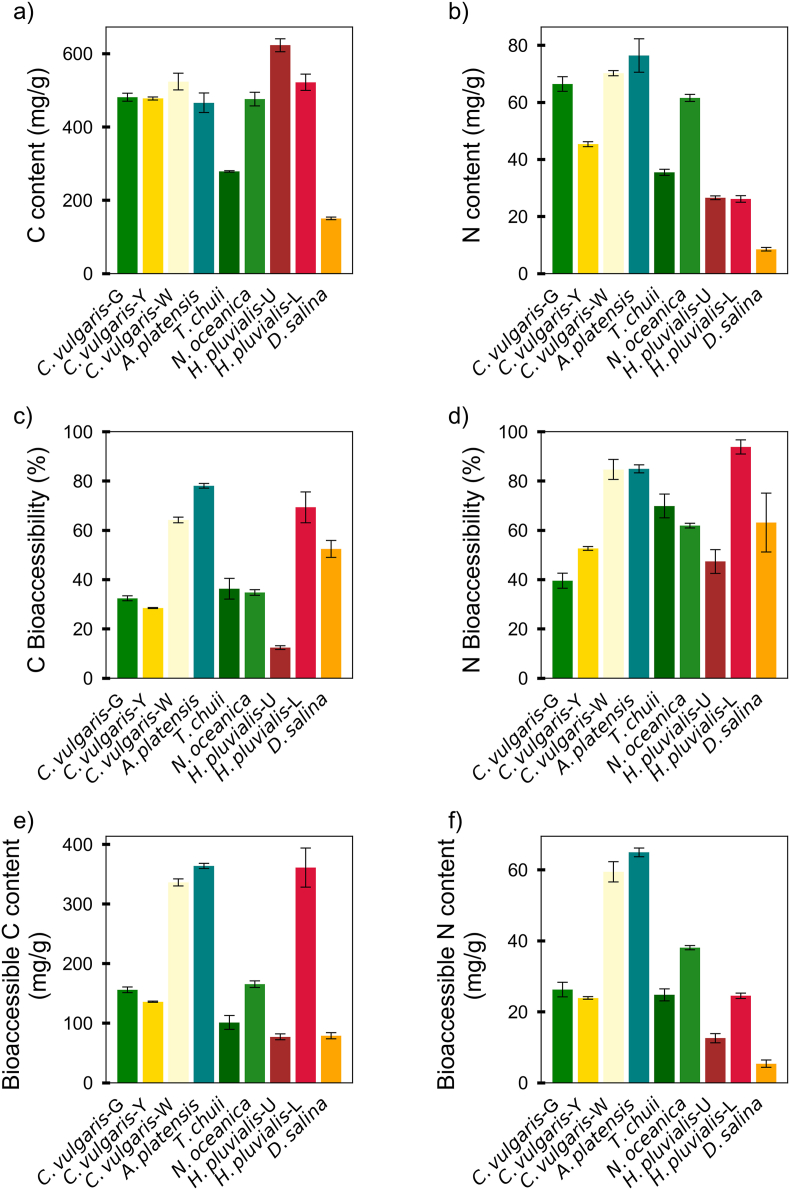
Biomass content, bioaccessibility, and bioaccessible content of carbon (a, c, e) and nitrogen (b, d, f) in 9 different microalgae species. ***Note****: From left to right, the abbreviations represent the following species: Chlorella vulgaris green (C. vulgaris-G); Chlorella vulgaris yellow (C. vulgaris-Y); Chlorella vulgaris white (C. vulgaris-W); Arthrospira platensis (A. platensis); Tetraselmis chuii (T. chuii); Nannochloropsis oceanica (N. oceanica); Haematococcus pluvialis (unlysed, H. pluvialis-U); Haematococcus pluvialis (lysed, H. pluvialis-L); and Dunaliella salina (D. salina). Data are presented as mean ± SD (n = 3).*

The total carbon (C) and nitrogen (N) content in the microalgae biomass varied across species (Fig. 4a and b). These differences in nitrogen contents may reflect variations in protein content among species, as nitrogen is a major constituent of amino acids and thus proteins. Similarly, high carbon content can be attributed to the accumulation of carbohydrates or lipids, both of which are rich in carbon atoms, with lipids consisting of long hydrocarbon chains.

Most microalgae exhibited C contents more than 450 mg/g, except for *D. salina* (150.3 ± 3.4 mg/g) and *T. chuii* (278.7 ± 2.1 mg/g), which showed lower values. For nitrogen (N) content, *A. platensis* displayed the highest concentration (76.4 ± 5.9 mg/g), followed by *C. vulgaris*-W (70.2 ± 0.9 mg/g) and *C. vulgaris*-G (66.4 ± 2.6 mg/g) (Fig. 4c). *D. salina* contained the lowest N content (8.5 ± 0.6 mg/g), while *H. pluvialis* species also exhibited relatively low nitrogen concentrations. The nitrogen level measured in *C. vulgaris*-G (∼66.4 mg/g) is consistent with the value reported (60.8 mg/g), indicating consistency with previous literature (Batista et al., 2013). Although the N content of *N. oceanica* (61.5 ± 1.2 mg/g) was slightly lower than that reported for *Nannochloropsis granulate* (70.1 mg/g), the results remain comparable considering species and strain variation (Tibbetts et al., 2015).

The bioaccessibility of C and N followed different trends across species (Fig. 4d and e). The C bioaccessibility was highest in *A. platensis* (78.1 ± 1.0 %), followed by *H. pluvialis*-L (69.3 ± 3.4 %) and *C. vulgaris*-W (64.2 ± 1.1 %). The lowest C bioaccessibility was observed in *H. pluvialis*-U (12.4 ± 0.8 %), despite its high total C content. For N, bioaccessibility showed high levels in most species. *H. pluvialis*-L had the highest N bioaccessibility (93.8 ± 2.9 %), followed by *C. vulgaris*-W (84.7 ± 4.1 %) and *A. platensis* (84.9 ± 1.6 %). The lowest N bioaccessibility was found in *D. salina* (63.1 ± 11.9 %), which also had the lower total N biomass content.

The absolute bioaccessible C and N content provides a clearer indication of how much carbon and nitrogen are potentially available for absorption (Fig. 4f and g). In terms of bioaccessible content, C bioaccessible content was highest in *A. platensis* (363.6 ± 4.4 mg/g), *H. pluvialis*-L (361.8 ± 32.7mg/g), and *C. vulgaris*-W (336.1 ± 6.0 mg/g), which were markedly higher than in other species. The remaining microalgae showed values between 77.2 and 165.5 mg/g. For nitrogen, the highest bioaccessible contents were found in *A. platensis* (64.9 ± 1.2 mg/g) and *C. vulgaris*-W (59.4 ± 2.9 mg/g). The lower N bioaccessible content was again observed in *D. salina* (5.4 ± 1.0 mg/g), in line with its low total N content.

Overall, *A. platensis*, *C. vulgaris*-W, and *H. pluvialis* species displayed high bioaccessible C and N content, suggesting their potential as good sources of organic carbon and nitrogenous compounds, such as carbohydrates, lipids, and proteins. The variations observed in bioaccessibility indicate differences in how carbon and nitrogen are retained and released among different species, which may influence their nutritional and functional properties.

### 3.5 Bioavailability of microalgae-derived iron

**Fig. 5:**
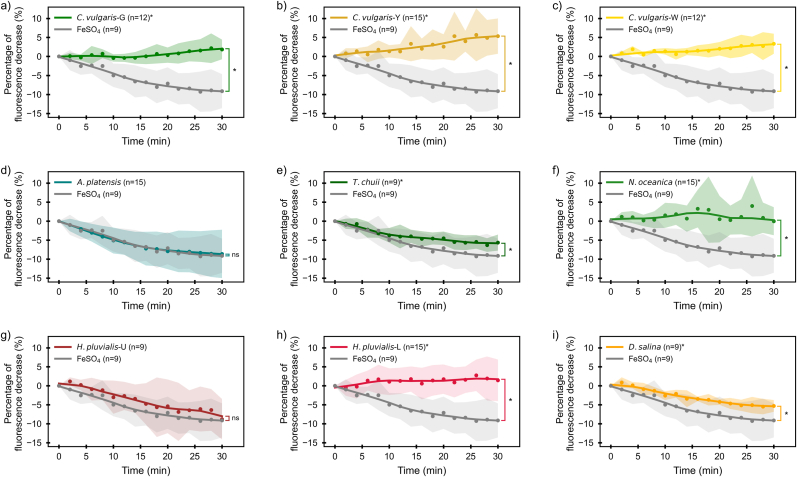
Real time iron absorption (percentage decline in fluorescence) compared to FeSO_4_. *Note: Lines represent locally weighted scatterplot smoothing (LOWESS) curves fitted to the mean values at each time point (using frac = 0.4). Shaded areas represent ± standard deviation (SD) from the mean. From a to i, the abbreviations represent the following species: Chlorella vulgaris green (C. vulgaris-G, n = 12)∗; Chlorella vulgaris yellow (C. vulgaris-Y, n = 15)∗; Chlorella vulgaris white (C. vulgaris-W, n = 12)∗; Arthrospira platensis (A. platensis, n = 15); Tetraselmis chuii (T. chuii, n = 9)∗; Nannochloropsis oceanica (N. oceanica, n = 15)∗; Haematococcus pluvialis (unlysed, H. pluvialis-U, n = 9); Haematococcus pluvialis (lysed, H. pluvialis-L, n = 15)∗; and Dunaliella salina (D. salina, n = 9)∗. Asterisks (∗) indicates statistically significant difference in iron bioavailability (p < 0.05) compared to FeSO*_*4*_ at 30 min*.*

Fe absorption by Caco-2 cells varied considerably among the 9 microalgae samples tested, as reflected by the percentage decrease in fluorescence over 30 min (Fig. 5) and 90 min (Fig. S1). These results represent the relative uptake of iron from digested microalgal biomass by intestinal epithelial cells under *in vitro* conditions. Since iron uptake is influenced by pH, absorption was evaluated under both acidic (pH 5.5) and neutral (pH 7.0) conditions to capture physiologically relevant scenarios (Wolff et al., 2018). As a positive control, freshly prepared FeSO_4_ was selected as the reference because of its well-established high solubility and rapid cellular uptake (Fanzaga et al., 2023). However, FeSO_4_ direct use as a dietary iron product is limited by issues such as poor stability during storage, gastrointestinal side effects, and interactions with other dietary components (Bloor et al., 2021).

**Fig. 6:**
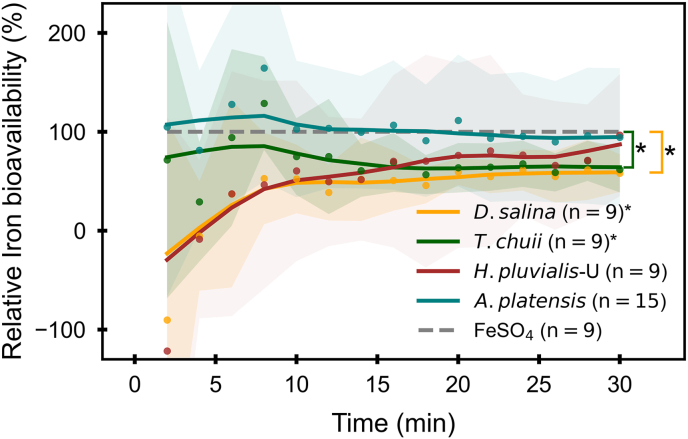
Time-resolved relative bioavailability of iron from 4 microalgae that exhibited measurable iron absorption, compared to FeSO_4_ over 30 min. ***Note:****Lines represent LOWESS-smoothed percentage fluorescence decrease curves over 30 min (using frac = 0.4). Shaded areas indicate ± standard deviation (SD) from the mean. D. salina (n = 9), T. chuii (n = 9), H. pluvialis-U (n = 9), and A. platensis (n = 15), showed measurable iron absorption in Caco-2 cells and were compared to FeSO*_*4*_*(n = 9). Asterisks (∗) indicate statistically significant differences in relative bioavailability compared to FeSO*_*4*_*(p* *<* *0.05, Welch's t-test). Significance bars denote the group-wise comparisons.* From top to bottom, the abbreviations represent the following species: *Dunaliella salina* (*D. salina*); *Tetraselmis chuii* (T. chuii); *Haematococcus pluvialis* (unlysed, *H. pluvialis*-U); *Arthrospira platensis* (*A. platensis*).

Among the 9 microalgal samples, 4 of them, including *T. chuii*, *H. pluvialis*-U, *D. salina* and **A*. platensis,* exhibited stable iron absorption under pH 5.5. The time-dependent absorption curves of these iron sources are comparable to that of the reference FeSO_4_, with **A*. platensis* showing an almost identical profile to FeSO_4_. The relative bioavailability of each microalgae species compared to FeSO_4_ (expressed as a percentage) is plotted over time (Fig. 6), highlighting differences in iron absorption from various microalgae sources. A relative bioavailability of 100 % indicates that iron absorption is equivalent to that of the reference FeSO_4_. The relative bioavailability of iron in species *H. pluvialis*-U and *D. salina* increased over time and stabilized after approximately 8 min. In contrast, Fe absorption from **A*. platensis* and *T. chuii* remained high at first (8 min), then decreased to a stable level. **A*. platensis* and *H. pluvialis*-U derived iron showed a similar bioavailability to FeSO_4_ (30 min), indicating promising potential as a highly bioavailable iron source.

At the endpoint of the absorption (30 min), *A. platensis* (mean value 94.5 ± 69.8 %; LOWESS-smoothed value 94.8 % ) and **H. pluvialis**-U (mean value 96.6 ± 61.6 %; LOWESS-smoothed value 87.3 %) showed no statistically significant difference compared to FeSO_4_ (*p* > 0.05), indicating similar bioavailability. Meanwhile, *T. chuii* (mean value 61.9 ± 23.2 %; LOWESS-smoothed value 64.2 %) and *D. salina* (mean value 58.1 ± 19.0 %; LOWESS-smoothed value 59.1 %) also demonstrated moderately high relative bioavailability, although the difference compared to FeSO_4_ remained significant. The relative bioavailability of microalgae-derived iron is remarkably high compared to other nutritional sources. For example, soybean seed ferritin has been reported to have remarkably lower bioavailability, whereas iron from fish and beef were reported to have significant lower bioavailability compared to FeSO_4_ with the presence of 1 mmol/L ascorbic acid (iron absorption enhancer) (Glahn et al., 1998; Lv et al., 2015). Microalgae, including *H. pluvialis*-U, **A*. platensis*, *T. chuii, and D.* salina, could serve as highly bioavailable iron sources based on the findings from this study.

At pH 7.0, no measurable iron uptake was observed in certain species (data not shown), demonstrating the critical role of pH in iron absorption. Previous studies have reported decreased iron uptake at elevated pH levels, particularly between pH 5.8 and 7.2, which is attributed to the proton-coupled mechanism of divalent metal transporter 1 (DMT 1) responsible for transporting divalent iron (Zhu et al., 2006; Salovaara et al., 2003).

No decline in the fluorescence signal was observed during the 90-min measurements for *C. vulgaris*-G, *C. vulgaris*-Y, *C. vulgaris*-W, *N. oceanica*, and *H. pluvialis*-L, indicating that no measurable iron uptake occurred in these species under the tested conditions. Microalgae *C. vulgaris*-G (83.4 ± 6.0 %) and *T. chuii* (0.51 ± 0.03 %) showed the highest and lowest bioaccessibility, respectively; however, their iron absorption exhibited opposite trends, even when equal concentrations of iron were used for both samples in the absorption measurements. *T. chuii* demonstrated good relative bioavailability (64.2 %), while no iron absorption was observed for *C. vulgaris*-G.

The poor iron absorption observed in certain microalgae species is likely governed by multiple mechanistic factors beyond iron content or bioaccessibility alone. First, the chemical form and redox state of iron plays a central role in intestinal uptake. Ferrous iron (Fe^2+^) can be directly transported by the DMT1, whereas ferric iron (Fe^3+^) exhibits low solubility and readily precipitates, thereby limiting its availability for absorption (Zhu et al., 2006). Second, the presence of endogenous reducing agents, particularly ascorbic acid, critically influences iron absorption (Lynch and Cook, 1980). Ascorbic acid enhances iron uptake through two complementary mechanisms: (i) by reducing Fe^3+^ to the more readily absorbable Fe^2+^ form, and (ii) by stabilizing iron in a soluble form by complexation, thereby reducing oxidation and precipitation at near-neutral pH (Salovaara et al., 2003; Lynch and Cook, 1980). In the present study, no detectable ascorbic acid was found in the digested microalgae samples (data not shown), suggesting limited reducing capacity after digestion to maintain iron in an absorbable form. Finally, iron absorption may be further suppressed by inhibitory compounds such as polyphenols and phytic acid, which are known to chelate iron and form insoluble complexes (Perfecto et al., 2017; He et al., 2008). These combined factors explain why high mineral content or high bioaccessibility does not necessarily result in high bioavailability.

Several universal strategies have been reported to improve iron bioavailability, including supplying reducing agents such as ascorbic acid to maintain iron in the ferrous state, reducing inhibitors like phytates and polyphenols through enzymatic or fermentation treatments, and promoting iron complexation with organic acids or peptides to enhance solubility (Piskin et al., 2022). Mildly acidic conditions and cell-disruption techniques may further facilitate the release and uptake of intracellular iron (Piskin et al., 2022).

## 4 Conclusion

This study comprehensively evaluated the content, bioaccessibility, and bioavailability of essential minerals, carbon, and nitrogen from 9 different microalgae biomass using standardized *in vitro* digestion and cellular absorption models. Results revealed notable interspecies differences in mineral content and bioaccessibility, demonstrating that high mineral content does not necessarily indicate high nutritional availability. For instance, *T. chuii* displayed the highest iron content yet extremely low bioaccessibility (0.5 %), whereas *C*. *vulgaris*-G exhibited high iron bioaccessibility (83.4 %) but negligible cellular iron uptake. *A*. *platensis* and unlysed *H. pluvialis* emerged as promising iron sources due to high bioavailability comparable to FeSO_4_. Additionally, bioaccessibility of Zn, Cu, Mg, Ca, P, carbon, and nitrogen varied markedly across species, whereas K remained consistently high (>90 %), underscoring the complexity of mineral availability. As hypothesized, microalgae exhibited clear inter-species differences in both mineral bioaccessibility and iron bioavailability, confirming that nutrient release and absorption potential are strongly species-dependent. Overall, these findings highlight the potential of microalgae as highly bioaccessible and bioavailable mineral sources. Future research should focus on optimizing biomass quality during production and harvest, incorporating washing steps to remove extracellular minerals, and conducting *in vivo* validation studies to strengthen the nutritional assessment.

## CRediT authorship contribution statement

Fengzheng Gao: Conceptualization, Methodology, Resources, Writing – review & editing, Supervision, Project administration, Funding acquisition. Shilei Chen: Investigation, Data curation, Visualization, Writing – original draft, Writing – review & editing. Xinyue Zhao: Formal analysis, Investigation (bioavailability), Data curation (bioavailability), Writing – original draft (bioavailability). Agon Besimi: Resources. Christophe Zeder: Methodology, Resources. Maria J. Barbosa: Resources, Writing – review & editing, Supervision. Ferdinand von Meyenn: Resources, Writing – review & editing, Supervision, Project administration, Funding acquisition. Alexander Mathys: Resources, Writing – review & editing, Supervision, Project administration, Funding acquisition.

## Funding

This project was funded and supported by the 10.13039/100000001Swiss National Science Foundation (Project No. 217292 and No. 228993), the 10.13039/501100003006ETH Zurich Foundation, and the Center for Innovation and Sustainability in Business (CISB) Foundation via the ETH Zurich Foundation. We thank the Bezos Earth Fund for their support to the Bezos Centre for Sustainable Protein at NUS.

## Declaration of competing interest

The authors declare that they have no known competing financial interests or personal relationships that could have appeared to influence the work reported in this paper.

## Data Availability

The datasets used in this study are available via the ETH Research Collection: https://doi.org/10.3929/ethz-c-000789102.
