# Supplementary material for: Bioaccessibility and bioavailability assessment of microalgae-derived minerals for human nutrition
Source: Curr Res Food Sci. 2026 Jan 6;12:101302. doi: 10.1016/j.crfs.2026.101302 (PMC12856855; doi:10.1016/j.crfs.2026.101302)
Supplement: Multimedia component 1 [file mmc1.docx]

Bioaccessibility and bioavailability assessment of microalgae-derived minerals for human nutrition Table S1. List of chemicals and reagents used in the study.

| **Reagent and Chemical** | **Catalog number** | **Manufacturer** | **Supplier** |
| --- | --- | --- | --- |
| Rabbit gastric extract | RGE15-1G | Lipolytech | Marseille, France |
| Pancreatin (porcine) | P7545-25G | Sigma-Aldrich | Merck, Germany |
| Bile extract | B3883-25G | Sigma-Aldrich | Merck, Germany |
| Hydrochloric acid (HCl) | 1.00317.1000 | Sigma-Aldrich | Merck, Germany |
| Nitric acid 65% | 1.00456.2510 | Sigma-Aldrich | Merck, Germany |
| TOC standard (1000 mg/L) | 76067-500ML-F | Sigma-Aldrich | Merck, Germany |
| NaNO_3_ | 221341 | Sigma-Aldrich | Merck, Germany |
| CaCl_2_(H_2_O)_2_ | C5080-500G | Sigma-Aldrich | Merck, Germany |
| FeSO_4_(H_2_O)_7_ | 215422 | Sigma-Aldrich | Merck, Germany |
| Calcein-AM | C3099 | Thermo Fisher Scientific | Waltham, USA |
| MEM NEAA (100×) | 11140-035 | Thermo Fisher Scientific | Waltham, USA |
| MEM (1×) | 11095-080 | Thermo Fisher Scientific | Waltham, USA |
| PBS pH 7.4 (1×) | 10010-015 | Thermo Fisher Scientific | Waltham, USA |
| HBSS (10×) | 14065-056 | Thermo Fisher Scientific | Waltham, USA |
| Pen-Strep | 15140-122 | Thermo Fisher Scientific | Waltham, USA |
| Trypsin-EDTA | 25200-056 | Thermo Fisher Scientific | Waltham, USA |

Table S2. List of equipment used in the study.

| **Equipment** | **Model** | **Manufacturer** | **Supplier** |
| --- | --- | --- | --- |
| Milli-Q water purifier | Synergy UV, ultrapure type 1 | Sigma-Aldrich | Merck, Germany |
| ICP-MS | iCAP RQ | Thermo Fisher Scientific | Waltham, USA |
| Microwave digestion system | TurboWave | MLS | Leutkirch, Germany |
| TOC/TN analyzer | TOC-L + TNM-L | Shimadzu | Kyoto, Japan |
| Plate reader | SynergyMx | BioTek Instruments | Winooski, USA |
| Dry bath incubator | ThermoMixer C | Eppendorf | Hamburg, Germany |
| Centrifuge | 5810R | Eppendorf | Hamburg, Germany |
| CO₂ incubator | - | Thermo Fisher Scientific | Waltham, USA |

Table S3. Mineral contents (mg/kg) of microalgae samples.

|  | **Fe (mg/kg)** | **Ca (mg/kg)** | **Zn (mg/kg)** | **Mg (mg/kg)** |
| --- | --- | --- | --- | --- |
| ***C. vulgaris*-G** | 75.85 ± 5.15 | 1651.74 ± 133.55 | 282.06 ± 16.91 | 1264.54 ± 37.88 |
| ***C. vulgaris*-Y** | 72.08 ± 3.24 | 4400.87 ± 257.97 | 239.82 ± 7.89 | 1228.18 ± 72.48 |
| ***C. vulgaris*-W** | 93.45 ± 2.22 | 3169.27 ± 56.66 | 206.25 ± 4.50 | 1427.82 ± 36.54 |
| ***A. platensis*** | 709.23 ± 138.85 | 12920.83 ± 1416.64 | 49.04 ± 11.23 | 2382.03 ± 220.99 |
| ***T. chuii*** | 3120.89 ± 255.55 | 24146.31 ± 664.41 | 67.40 ± 9.51 | 9435.69 ± 47.28 |
| ***N. oceanica*** | 382.84 ± 8.27 | 3937.41 ± 45.34 | 20.03 ± 1.99 | 7198.12 ± 32.92 |
| ***H. pluvialis*-U** | 156.03 ± 30.23 | 516.93 ± 69.13 | 12.66 ± 6.31 | 422.97 ± 41.84 |
| ***H. pluvialis*-L** | 434.51 ± 16.78 | 1537.32 ± 353.75 | 12.09 ± 0.01 | 379.20 ± 43.92 |
| ***D. salina*** | 441.82 ± 59.19 | 10359.55 ± 587.62 | 33.92 ± 9.92 | 15245.92 ± 1072.98 |

|  | **Cu (mg/kg)** | **Mn (mg/kg)** | **P (mg/kg)** | **K (mg/kg)** |
| --- | --- | --- | --- | --- |
| ***C. vulgaris*-G** | 2.34 ± 0.10 | 112.34 ± 2.66 | 11511.72 ± 660.74 | 61235.94 ± 5627.90 |
| ***C. vulgaris*-Y** | 1.91 ± 0.05 | 69.35 ± 3.47 | 10516.63 ± 531.22 | 34288.97 ± 3686.41 |
| ***C. vulgaris*-W** | 2.69 ± 0.06 | 76.09 ± 0.86 | 13917.93 ± 720.72 | 47945.94 ± 9454.88 |
| ***A. platensis*** | 2.11 ± 0.18 | 56.16 ± 9.58 | 6204.92 ± 1712.31 | 54677.41 ± 7313.80 |
| ***T. chuii*** | 3.25 ± 0.14 | 79.59 ± 3.53 | 11325.73 ± 678.01 | 57901.98 ± 2422.16 |
| ***N. oceanica*** | 1.81 ± 0.08 | 40.77 ± 0.46 | 9078.74 ± 294.74 | 37178.64 ± 3195.63 |
| ***H. pluvialis*-U** | 1.49 ± 0.18 | 13.61 ± 1.06 | 1660.31 ± 376.11 | 9042.08 ± 3869.14 |
| ***H. pluvialis*-L** | 1.34 ± 0.10 | 23.12 ± 1.22 | 1495.05 ± 346.77 | 26585.12 ± 2270.93 |
| ***D. salina*** | 2.97 ± 0.17 | 26.76 ± 2.16 | 942.62 ± 147.20 | 33089.40 ± 7027.11 |

*Note: The abbreviations, listed from top to bottom, correspond to the following species: Chlorella vulgaris green (C. vulgaris-G); Chlorella vulgaris yellow (C. vulgaris-Y); Chlorella vulgaris white (C. vulgaris‑W); Arthrospira platensis (A. platensis); Tetraselmis chuii (T. chuii); Nannochloropsis oceanica (N. oceanica); Haematococcus pluvialis (unlysed, H. pluvialis-U); Haematococcus pluvialis (lysed, H. pluvialis-L); and Dunaliella salina (D. salina). Data are presented as mean ± SD (n = 3).*

Table S4. Ash content (%) of microalgae samples.

|  | **Ash content (%)** |
| --- | --- |
| *C. vulgaris*-G | 7.55 ± 0.11 |
| *C. vulgaris*-Y | 5.10 ± 0.16 |
| *C. vulgaris*-W | 8.25 ± 0.07 |
| *A. platensis* | 14.46 ± 0.12 |
| *T. chuii* | 30.99 ± 0.09 |
| *N. oceanica* | 23.42 ± 0.15 |
| *H. pluvialis*-U | 2.11 ± 0.56 |
| *H. pluvialis*-L | 2.15 ± 0.21 |
| *D. salina* | 52.67 ± 0.26 |

*Note: The abbreviations, listed from top to bottom, correspond to the following species: Chlorella vulgaris green (C. vulgaris-G); Chlorella vulgaris yellow (C. vulgaris-Y); Chlorella vulgaris white (C. vulgaris‑W); Arthrospira platensis (A. platensis); Tetraselmis chuii (T. chuii); Nannochloropsis oceanica (N. oceanica); Haematococcus pluvialis (unlysed, H. pluvialis-U); Haematococcus pluvialis (lysed, H. pluvialis-L); and Dunaliella salina (D. salina). Data are presented as mean ± SD (n = 3).*

Table S5. Mineral Bioaccessibility (%) of microalgae samples.

|  | **Fe** | **Ca** | **Zn** | **Mg** | **Cu** | **Mn** | **P** | **K** |
| --- | --- | --- | --- | --- | --- | --- | --- | --- |
| ***C. vulgaris*-G** | 83.43 ± 6.02 | 72.51 ± 4.44 | 62.09 ± 6.78 | 84.79 ± 0.63 | 71.87 ± 3.53 | 84.25 ± 0.51 | 72.48 ± 3.72 | 97.97 ± 1.54 |
| ***C. vulgaris*-Y** | 53.36 ± 1.56 | 50.92 ± 3.84 | 51.89 ± 7.08 | 68.00 ± 1.68 | 80.21 ± 0.47 | 55.93 ± 4.29 | 61.13 ± 2.59 | 93.78 ± 1.99 |
| ***C. vulgaris*-W** | 53.59 ± 2.69 | 55.13 ± 0.31 | 57.05 ± 4.56 | 79.11 ± 0.78 | 76.05 ± 5.63 | 66.12 ± 4.54 | 69.96 ± 3.88 | 98.49 ± 1.66 |
| ***A. platensis*** | 9.55 ± 1.69 | 70.19 ± 7.50 | 62.17 ± 4.48 | 88.28 ± 5.44 | 86.96 ± 2.92 | 34.62 ± 8.13 | 52.22 ± 17.50 | 107.92 ± 5.49 |
| ***T. chuii*** | 0.51 ± 0.03 | 33.67 ± 1.86 | - | 76.22 ± 1.56 | 48.04 ± 1.34 | 6.75 ± 0.88 | 23.39 ± 2.92 | 97.28 ± 1.23 |
| ***N. oceanica*** | 5.11 ± 0.22 | 61.00 ± 2.47 | - | 89.03 ± 1.72 | 7.49 ± 1.87 | 42.84 ± 1.21 | 53.33 ± 0.82 | 105.03 ± 4.88 |
| ***H. pluvialis*-U** | 17.72 ± 3.98 | 68.77 ± 9.43 | - | 70.57 ± 5.65 | 59.19 ± 7.68 | 57.27 ± 3.01 | 55.04 ± 5.58 | 118.12 ± 15.88 |
| ***H. pluvialis*-L** | 45.79 ± 2.47 | -3.17 ± 4.52 | - | 78.47 ± 5.26 | 89.75 ± 6.30 | 67.27 ± 1.29 | 108.39 ± 18.55 | 104.75 ± 5.55 |
| ***D. salina*** | 7.67 ± 1.61 | 82.26 ± 6.61 | - | 92.22 ± 5.50 | 79.15 ± 4.12 | 45.33 ± 8.31 | 9.65 ± 17.02 | 91.05 ± 6.93 |

*Note: The abbreviations, listed from top to bottom, correspond to the following species: Chlorella vulgaris green (C. vulgaris-G); Chlorella vulgaris yellow (C. vulgaris-Y); Chlorella vulgaris white (C. vulgaris‑W); Arthrospira platensis (A. platensis); Tetraselmis chuii (T. chuii); Nannochloropsis oceanica (N. oceanica); Haematococcus pluvialis (unlysed, H. pluvialis-U); Haematococcus pluvialis (lysed, H. pluvialis-L); and Dunaliella salina (D. salina). Data are presented as mean ± SD (n = 3).*

Table S6. Mineral bioaccessible contents (mg/kg) of microalgae samples.

|  | **Fe (mg/kg)** | **Ca (mg/kg)** | **Zn (mg/kg)** | **Mg (mg/kg)** |
| --- | --- | --- | --- | --- |
| ***C. vulgaris*-G** | 63.28 ± 4.57 | 1197.68 ± 73.34 | 175.13 ± 19.12 | 1072.20 ± 7.97 |
| ***C. vulgaris*-Y** | 38.46 ± 1.12 | 2240.48 ± 168.99 | 124.44 ± 16.98 | 835.16 ± 20.63 |
| ***C. vulgaris*-W** | 50.07 ± 2.51 | 1747.22 ± 9.82 | 117.66 ± 9.40 | 1129.55 ± 11.14 |
| ***A. platensis*** | 67.73 ± 11.99 | 9069.13 ± 969.06 | 30.49 ± 2.20 | 2102.85 ± 129.58 |
| ***T. chuii*** | 16.07 ± 0.95 | 8130.06 ± 449.12 | - | 7191.89 ± 147.20 |
| ***N. oceanica*** | 19.56 ± 0.84 | 2401.82 ± 97.25 | - | 6408.48 ± 123.81 |
| ***H. pluvialis*-U** | 27.65 ± 6.19 | 355.49 ± 48.69 | - | 298.49 ± 23.90 |
| ***H. pluvialis*-L** | 198.96 ± 10.73 | - | - | 297.55 ± 19.95 |
| ***D. salina*** | 33.89 ± 7.11 | 8521.76 ± 684.77 | - | 14059.79 ± 838.53 |

|  | **Cu (mg/kg)** | **Mn (mg/kg)** | **P (mg/kg)** | **K (mg/kg)** |
| --- | --- | --- | --- | --- |
| ***C. vulgaris*-G** | 1.69 ± 0.08 | 94.64 ± 0.57 | 8343.70 ± 428.24 | 59992.86 ± 943.03 |
| ***C. vulgaris*-Y** | 1.53 ± 0.01 | 38.79 ± 2.98 | 6428.81 ± 272.38 | 32156.19 ± 682.35 |
| ***C. vulgaris*-W** | 2.04 ± 0.15 | 50.31 ± 3.45 | 9736.99 ± 540.02 | 47221.95 ± 795.90 |
| ***A. platensis*** | 1.84 ± 0.06 | 19.44 ± 4.57 | 3302.26 ± 1085.86 | 59007.86 ± 3001.79 |
| ***T. chuii*** | 1.56 ± 0.04 | 5.37 ± 0.70 | 2649.09 ± 330.71 | 56327.05 ± 706.40 |
| ***N. oceanica*** | 0.14 ± 0.03 | 17.46 ± 0.49 | 4841.69 ± 74.45 | 39048.73 ± 1814.32 |
| ***H. pluvialis*-U** | 0.88 ± 0.11 | 7.79 ± 0.41 | 913.84 ± 92.65 | 10680.50 ± 1435.88 |
| ***H. pluvialis*-L** | 1.20 ± 0.08 | 15.55 ± 0.30 | 1620.48 ± 277.33 | 27847.92 ± 1475.47 |
| ***D. salina*** | 2.35 ± 0.12 | 12.13 ± 2.22 | 90.96 ± 160.43 | 30127.90 ± 2293.10 |

*Note: The abbreviations, listed from top to bottom, correspond to the following species: Chlorella vulgaris green (C. vulgaris-G); Chlorella vulgaris yellow (C. vulgaris-Y); Chlorella vulgaris white (C. vulgaris‑W); Arthrospira platensis (A. platensis); Tetraselmis chuii (T. chuii); Nannochloropsis oceanica (N. oceanica); Haematococcus pluvialis (unlysed, H. pluvialis-U); Haematococcus pluvialis (lysed, H. pluvialis-L); and Dunaliella salina (D. salina). Data are presented as mean ± SD (n = 3).*


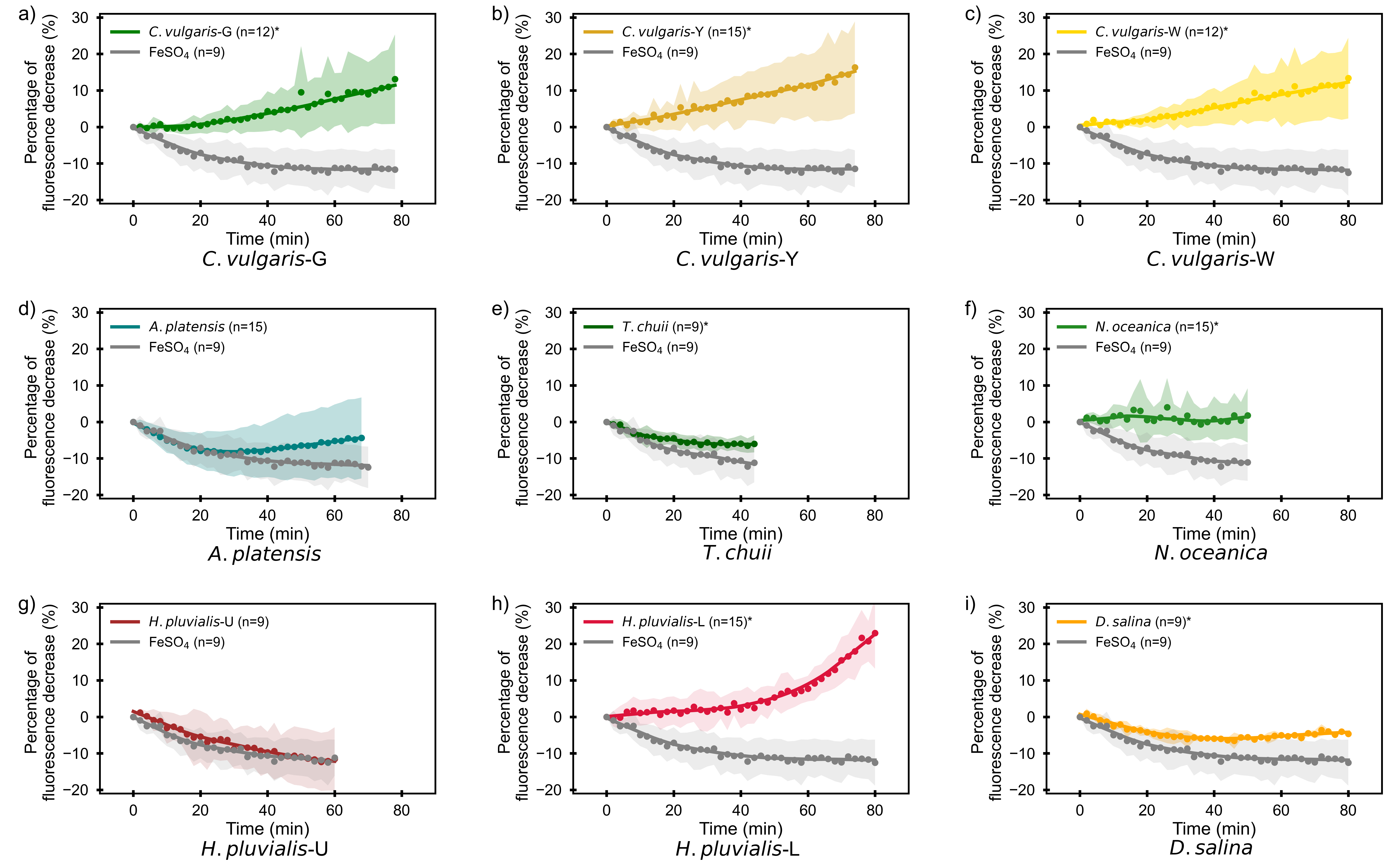


**Figure S1.** Real time iron absorption (percentage decline in fluorescence) compared to FeSO_4_.

Note: Lines represent locally weighted scatterplot smoothing (LOWESS) curves fitted to the mean values at each time point (using frac = 0.4). Shaded areas represent ±1 standard deviation (SD) from the mean. From a to i, the abbreviations represent the following species: Chlorella vulgaris green (C. vulgaris-G, n = 12); Chlorella vulgaris yellow (C. vulgaris-Y, n = 15); Chlorella vulgaris white (C. vulgaris-W, n = 12); Arthrospira platensis (A. platensis, n = 15); Tetraselmis chuii (T. chuii, n = 9); Nannochloropsis oceanica (N. oceanica, n = 15); Haematococcus pluvialis (unlysed, H. pluvialis-U, n = 9); Haematococcus pluvialis (lysed, H. pluvialis-L, n = 15); and Dunaliella salina (D. salina, n = 9).
